# The effect of surface charge on nonspecific uptake and cytotoxicity of CdSe/ZnS core/shell quantum dots

**DOI:** 10.3762/bjnano.6.26

**Published:** 2015-01-26

**Authors:** Vladimir V Breus, Anna Pietuch, Marco Tarantola, Thomas Basché, Andreas Janshoff

**Affiliations:** 1 Institute of Physical Chemistry, Johannes Gutenberg University Mainz, Jakob-Welder-Weg 11, 55128 Mainz, Germany; 2 Institute of Physical Chemistry, University of Goettingen, Tammannstr. 6, 37077 Goettingen, Germany; 3 Max-Planck-Institute for Dynamics and Self-Organization (MPIDS), Laboratory for Fluid Dynamics, Pattern Formation and Biocomplexity, Am Fassberg 17, 37077 Goettingen, Germany

**Keywords:** biocompatibility, CdSe/ZnS, cytotoxicity, ECIS, quantum dots, single-particle tracking

## Abstract

In this work, cytotoxicity and cellular impedance response was compared for CdSe/ZnS core/shell quantum dots (QDs) with positively charged cysteamine–QDs, negatively charged dihydrolipoic acid–QDs and zwitterionic D-penicillamine–QDs exposed to canine kidney MDCKII cells. Pretreatment of cells with pharmacological inhibitors suggested that the uptake of nanoparticles was largely due to receptor-independent pathways or spontaneous entry for carboxylated and zwitterionic QDs, while for amine-functionalized particles involvement of cholesterol-enriched membrane domains is conceivable. Cysteamine–QDs were found to be the least cytotoxic, while D-penicillamine–QDs reduced the mitochondrial activity of MDCKII by 20–25%. Although the cell vitality appeared unaffected (assessed from the changes in mitochondrial activity using a classical MTS assay after 24 h of exposure), the binding of QDs to the cellular interior and their movement across cytoskeletal filaments (captured and characterized by single-particle tracking), was shown to compromise the integrity of the cytoskeletal and plasma membrane dynamics, as evidenced by electric cell–substrate impedance sensing.

## Introduction

Quantum dots (QDs) are advantageous tools for fluorescent labeling that have gained major attention over the past decade from various fields of application in the life sciences [1–6]. They are typically brighter than conventional organic dyes, much more resistant against photobleaching and their size-dependent optical properties can easily be tuned over the entire range of the visible spectrum. Due to their hazardous inorganic content, together with their small size and considerably large surface area available for enzymatic degradation, potential toxic effects are of great concern [7]. High levels of cytotoxicity resulting from CdSe and CdTe QD exposure to cultured cells was attributed to the presence of Cd^2+^ ions during the initial stages of synthesis or during in situ release, resulting in mitochondrial damage and oxidative stress [8–9]. The isolation of toxic core contents by coating the CdSe nanocrystals with a few monolayers of the nontoxic semiconducting material ZnS was found to reduce or even completely abolish cytotoxicity [9–10] Moreover, it was also suggested that the toxic effects of QDs depend more on the type and integrity of the surface coatings rather than the inorganic nanocrystal itself [11]. Various functionalization strategies have been employed in order to increase the stability of the surface ligand shell and to reduce the cytotoxicity of QDs, such as the use of cross-linked polymer coatings [10,12–13] or encapsulation in a silica shell [14–16]. These approaches, however, also increase the overall size of the nanoparticles, which may alter their uptake mechanism and limit some of their applications. Due to their complex structure and different potential sources of damage (e.g., air and photooxidation, opsonization and enzymatic degradation, mechanical damage, etc.), means for effective characterization of the cytotoxicity are still to be defined. The majority of reports on QD cytotoxicity use conventional MTS and MTT assays [8–9,11,17–19], live/dead reagents and viability controls [14,20] to quantify the QD-impaired damage such as ROS production and mitochondrial membrane permeability assays. We demonstrate that these methods, however, can overlook other more subtle impacts on cell viability and metabolism caused by binding of QDs to cellular compartments, without release of Cd^2+^ ions.

In the present study, we use a noninvasive and label-free impedance setup to quantify the cytotoxic effects of QDs on the viability of MDCKII cells in combination with single-particle tracking employing a wide-field fluorescence microscope [21]. The impact of QDs with different surface charges is characterized at the initial stages after exposure to cells, the stage at which classical cytotoxicity tests do not recognize QD-induced damage. We expose the cells to solutions of CdSe/ZnS core/shell QDs, functionalized with cysteamine (CA), dihydrolipoic acid (DHLA) and D-penicillamine (DPA), producing positively-charged, negatively-charged and zwitterionic particle surfaces, respectively. Electric cell–substrate impedance sensing (ECIS), which was first described by Giaever and Keese [22], offers a versatile and noninvasive means to monitor cellular adhesion and motility on a subsecond timescale [23–24]. After adhesion and spreading on a gold electrode surface, cells behave as insulators, blocking the current flow at the applied frequency of 4 kHz and thereby enhancing the impedance signal. Time-resolved measurements of the cellular impedance signal provide information about the behavior of the adherent epithelial cell monolayer and its response upon nanoparticle exposure [25–27]. Furthermore, we combined ECIS with single-particle tracking, which was previously used to follow the intracellular pathway of Tat peptide-conjugated QDs in living cells [28], transport of QD-labelled monoclonal anti-HER2 antibody in mice [29], specific recognition of avidin-CD14 receptor by biotinylated QDs [30], or to observe the movement of single, streptavidin-coated QDs along microtubules [31].

## Results and Discussion

**Scheme 1:**
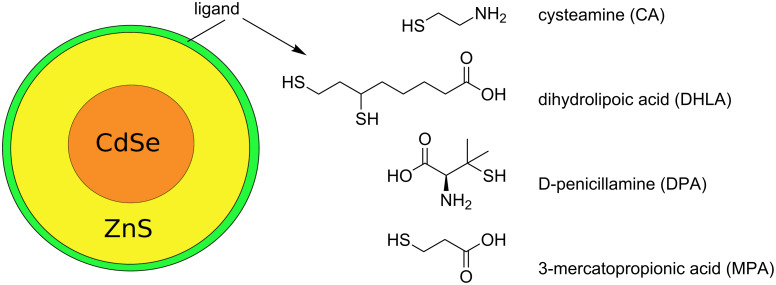
CdSe/ZnS quantum dot with the ligands used in this study.

ECIS and the MTS assay were used to evaluate the viability of MDCKII cells exposed to CdSe/ZnS QDs functionalized with positively-charged CA ligands, negatively-charged DHLA- or MPA-ligands, and zwitterionic DPA ligands (Scheme 1).

At physiological pH 7.4, CA–QDs exhibit high aggregation rates in aqueous solutions, with hydrodynamic diameters 4–5 times larger than those of the as-prepared particles with the initial coating in organic solution [11]. In contrast, DHLA–QDs and DPA–QDs show minimal aggregation due to the low p*K*_a_ value (4.73) of the DHLA carboxylic group [32] and the colloidal stability over a wide pH range of the zwitterionic coating, respectively [33]. In order to clarify whether the CA–QDs and DHLA–QDs affect the cells differently due to their surface charge or the aggregation rate (which reduces the effective concentration of single QDs in solution), we studied an alternative, carboxylated, MPA-coated preparation, which is only partially deprotonated at pH 7.4.

**Figure 1:**
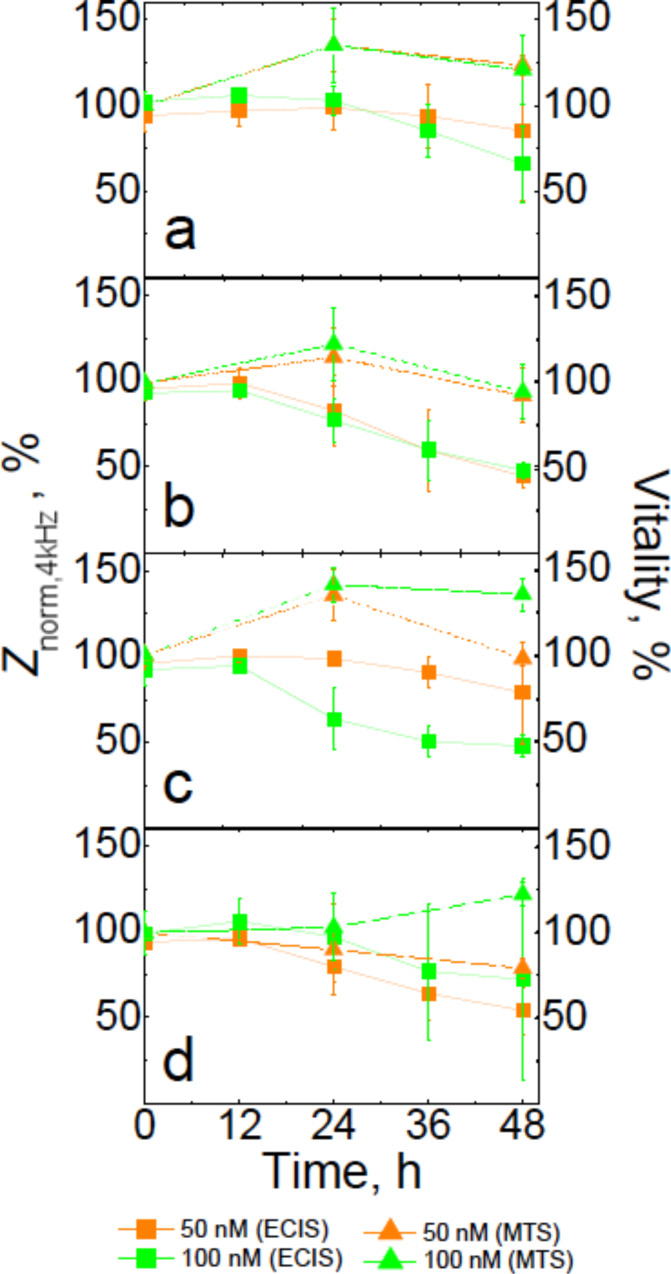
Normalized impedance (filled square) and mitochondrial activity (filled triangle) of MDCKII cells as a function of exposure time to (a) CA–QDs, (b) DHLA–QDs, (c) MPA–QDs and (d) DPA–QDs. Orange and green lines correspond to 50 and 100 nM concentrations of QDs, respectively.

We found that independent of the type and concentration of CdSe/ZnS QDs, the MDCKII cells display a significant impedance reduction after 48 h compared to untreated cells (Figure 1), indicating that the cell dynamics as well as the integrity of plasma membrane is compromised by QDs. Furthermore, the impact of the nanoparticles on the cells occurred mostly during hours 12–36, suggesting no immediate toxicity caused by QDs. For negatively charged DHLA–QDs and zwitterionic DPA–QDs, the decrease in the cellular impedance was the most pronounced, reduced to 50% of the initial value. 50 nM solutions of CA–QDs and MPA–QDs (for which limited colloidal stability was expected) both had the smallest effect on MDCKII impedance, while at higher concentrations, the impact of MPA–QDs was considerably higher than for CA–QDs (Figure 1a,c). These results suggest that the effect of positively charged CA–QDs on cell impedance is less pronounced than for DHLA–QDs and DPA–QDs, most likely due to a different interaction mechanism rather than a higher aggregation rate. Unlike the ECIS measurements, the MTS assay did not reveal a significant negative impact of QDs on the viability of cells (Figure 1).

**Figure 2:**
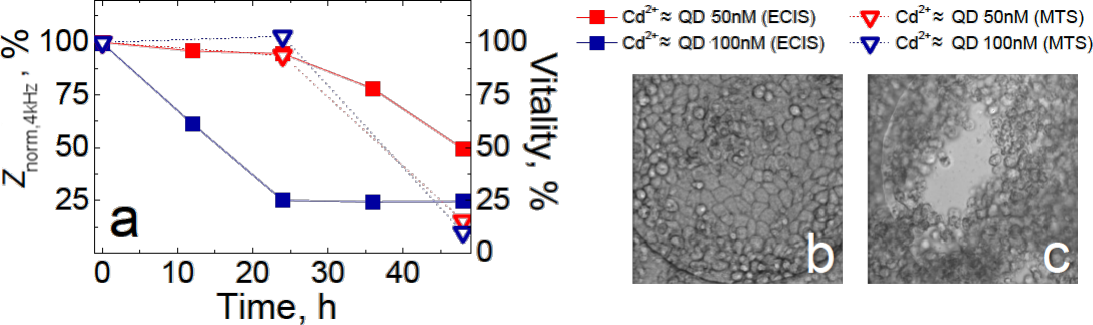
(a) Normalized impedance and mitochondrial activity of MDCKII cells as a function of exposure time to cadmium acetate stock solutions with a Cd^2+^ content corresponding to that of the 50 and 100 nM QD solutions. Images of the ECIS electrode with untreated cells (b), and after 48 h of exposure to cadmium acetate (c).

Overall, we observed no reduction of mitochondrial activity for cells exposed to both 50 and 100 nM solutions of positively- and negatively-charged QDs within 48 h, and an ≈10–20% loss of vitality after interaction with 50 nM zwitterionic QDs for 24 and 48 h. The 20–40% gain in mitochondrial activity of MDCKII cells observed for cells after 24 h of interaction with QDs could be attributed to an increase in cell proliferation, which was confirmed by fluorescence microscopy (Supporting Information File 1, Figure S1). We observed a small fraction of abnormally large cells (twice as large as the normal size), and cells with two nuclei after exposure to the 50 nM solutions of QDs (Supporting Information File 1, Figure S1). Thus, even though the MTS assay indicated that the cells were still healthy and retained their initial level of vitality within 24 h of exposure to QDs, the particles affected the dynamics and mechanics of MDCKII cell division and changed the properties of the plasma membrane. In Figure 2a, impedance changes and vitality of MDCKII cells upon 48 h exposure to various cadmium acetate stock solutions are compared.

Once again, ECIS was found to be much more sensitive in monitoring the cadmium cytotoxicity than the MTS test. The ECIS results showed a dramatic reduction of MDCKII impedance within the first 24 h of exposure to cadmium acetate with concentration corresponding to the Cd^2+^ content in 100 nM CdSe QD solutions (Figure 2a). After 48 h of interaction, both stock solutions of cadmium acetate caused a substantial impedance decrease and an almost complete reduction of MDCKII vitality, along with disruption of the confluent cell layer (Figure 2a–c). Comparing the results presented in Figure 1 and Figure 2, it can be assumed that the decomposition of the CdSe core protected by the ZnS shell did not occur within 48 h of exposure to cells, and thus is likely not the main source of QD-induced damage to a cell (in this time regime).

**Figure 3:**
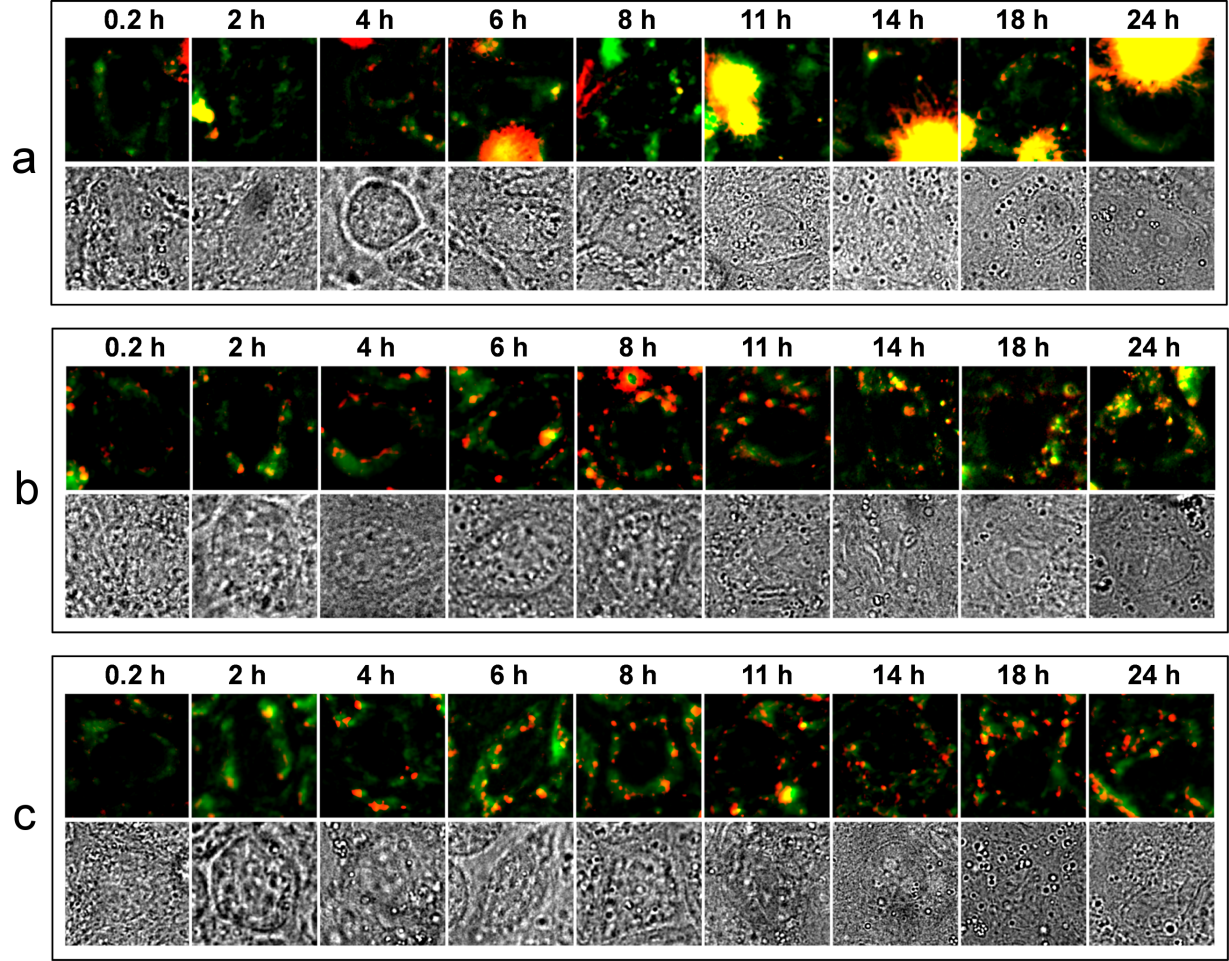
Composite images of QD fluorescence (red) and cell autofluorescence (green) together with corresponding transmission bright-field micrographs. The fluorescence signals from QDs and cells are extracted from the overall fluorescent signal by applying standard deviation (for QDs) and averaging (for cells), post-processing algorithms to a 20-frame image sequence obtained during each measurement.

In order to estimate the extent of QD internalization within MDCKII cells upon exposure, and to investigate the kinetics of their nonspecific interaction, a series of fluorescence images of different areas of MDCKII confluent layers was acquired during 24 hours of exposure to 10 nM QD solutions, as illustrated in Figure 3. Each fluorescence image is presented as an overlay of a standard deviation (in red) and an average (in green) of 20 subsequent 4 s-exposure scans, as explained in Supporting Information File 1, Figure S4. Thus, the red channel corresponds to the enhanced fluorescent signal due to moving species in the cell, while the green channel corresponds to the amplified cell autofluorescence (Supporting Information File 1, Figure S4). Additionally, the corresponding transmission bright-field micrographs are shown below each fluorescence image (Figure 3).

**Figure 4:**
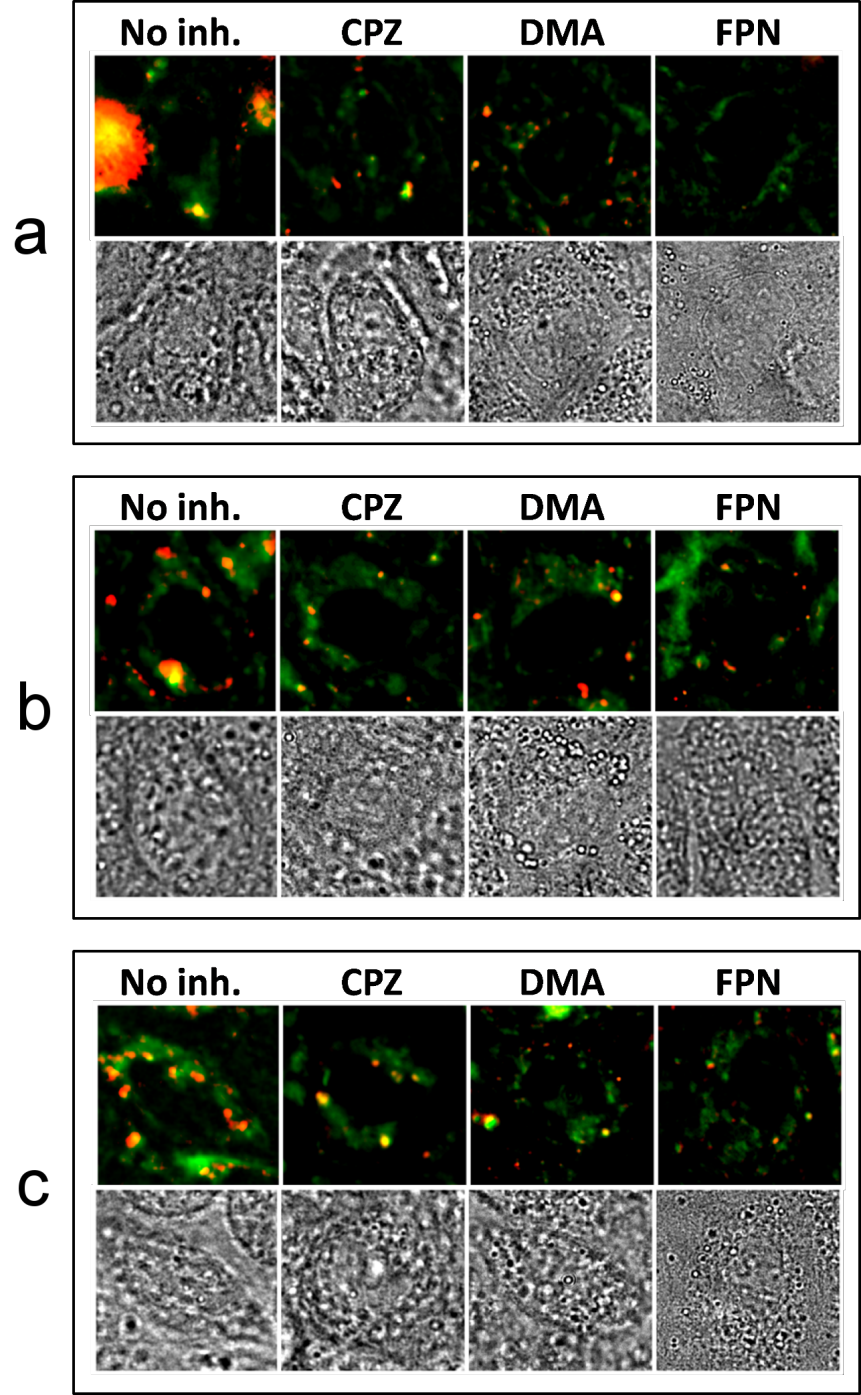
Composite images of QD fluorescence (red) and cell autofluorescence (green) together with corresponding transmission micrographs. Fluorescence signals from QDs and cells are extracted from the overall fluorescent signal by applying standard deviation (for QDs) and averaging (for cells), post-processing algorithms to a 20-frame image sequence obtained during each measurement. CPZ: chlorpromazine (inhibitior of clathrin-mediated endocytosis), DMA: dimethylamyloride hydrochloride (inhibitor of macropinocytosis), FPN: filipin III (inhibitor of caveolin-mediated endocytosis).

For CA–QDs, a strong out-of-focus fluorescence from large aggregates floating in solution was observed. The aggregates were immediately present after addition of QDs and did not bind to the cells. Within the first 4–6 h of exposure, primarily single CA–QDs and very small aggregates attached to the cells and appeared as small but bright fluorescent spots on the membrane and within the cell (Figure 3a). The uptake of single CA–QDs saturated after 11 h, while the number of large CA–QD-aggregates bound to the confluent MDCKII layer evidently increased from 8 to 24 h after addition of the particles (Figure 3a). The growth of CA–QDs aggregates over time could be caused by opsonization (e.g., the adsorption of proteins from the cell culture medium on the nanoparticles). Serum-induced aggregation was reported earlier for CdSe/ZnS QDs with cationic charge [19]. Some very large fluorescent aggregates can be seen after longer incubation times (Figure 3a) and were also visible in the corresponding transmission micrograph. They were identified as debris of dead cells labeled by QDs and floating above the plane of the confluent MDCKII layer. Figure S2a,b in Supporting Information File 1 demonstrates that the low mean fluorescence intensity of the nucleus areas in the center of cells in Figure 3a and Figure 4a, did not change over the course of the 24 h interaction period of CA–QDs and MDCKII cells.

For DHLA–QDs, a weak signal from few single particles attracted to the membrane was observed immediately after addition of the QD solution and after 2–4 h of exposure in the membrane-enclosed interior, too (Figure 3b). By changing focal plane, more QDs deeper within the MDCKII interior were found after 6–8 h of incubation (Figure 3b). Unlike amine-functionalized CA–QDs, negatively-charged DHLA-coated particles evinced only an insignificant aggregation rate, as discussed above. After 11 h of exposure to DHLA–QDs, few larger fluorescent spots appeared on the surface of the MDCKII cell layer (Figure 3b). Since the MTS assay showed no significant decrease of mitochondrial activity for the same or even higher concentrations of DHLA–QDs on MDCKII cells after 24 h (Figure 1b), we suggest that DHLA–QDs did not cause cell death, but instead labeled the interior of the cell, especially at the cell periphery. For longer interaction times, DHLA–QDs induced a small increase in the fluorescent signal in the nuclei (see Supporting Information File 1, Figure S2b).

Finally, zwitterionic DPA-coated QDs showed quite similar interaction kinetics as negatively-charged DHLA–QDs (Figure 3b,c). DPA–QDs that interacted with cells appeared largely as single fluorescence spots on the membrane and within the cell interior during the first 6 h after addition of QDs. For longer exposure times, the cell interior close to the nucleus displays more particles (Figure 3c).

In order to elucidate the mechanism of QD internalization by MDCKII cells, we studied nonspecific interactions between QDs and inhibitor-treated MDCK II cells after 6 h of exposure. We used chlorpromazine to inhibit the formation of clathrin-coated pits through the assembly of clathrin and AP2 adaptor complexes in the endosomal membranes, 5-(*N*,*N*-dimethyl)amiloride hydrochloride (DMA). This prohibited macropinocytosis by blocking amiloride-sensitive Na^+^/H^+^ exchange, and filipin III, which interacts with cholesterol in membranes and is reported to prevent the formation of caveolae [34]. In these experiments (Figure 4), we exposed cells to QDs for only 6 h, which is sufficient time for QDs to interact with the cell but well below adaption times of the cells to the inhibitor effects. Comparing the obtained series of scans with those of QDs exposed to untreated cells in Figure 3 and Figure 4 (first column), we found that MDCKII cells (after treatment with chlorpromazine and DMA) still exhibited uptake of a significant amount of positively-charged CA–QDs after 6 h, while cells exposed to filipin did not show any interaction with single QDs (Figure 4a). It is conceivable that cholesterol-enriched domains in the plasma membrane might be responsible for the binding of the CA–QDs. We also observed vesicle formation from the plasma membrane during 2–4 hours of exposure of cells to CA–QDs. A series of frames shown in Supporting Information File 1, Figure S3 illustrates the CA–QD-induced endosome shaping from the MDCKII cell membrane into the cellular interior after 2 h of interaction. The fluorescent spot corresponding to the formed vesicle is approximate 600 nm in diameter (Supporting Information File 1, Figure S3). However, due to the point spread function of the microscope, we can assume that the actual size of the endosome is smaller. Accordingly, the observed vesicles might correspond to caveolae (*d* = 50–100 nm), rather than large macropinosomes (*d* = 0.5–5 μm), which should lead to fluorescence spots much larger than 600 nm.

In contrast to the case of CA–QDs, exposure of cells to DHLA- and DPA-coated QDs still resulted in a considerable uptake (Figure 4b,c), largely suggesting a spontaneous entry, rather than a receptor-mediated uptake. Even though the DMA-treated cells still display interaction with DHLA–QDs and DPA–QDs, we cannot exclude that macropinocytosis was responsible for particle uptake, since all known pharmacological inhibitors have only limited efficiency for this receptor-independent endocytic pathway [35].

**Figure 5:**
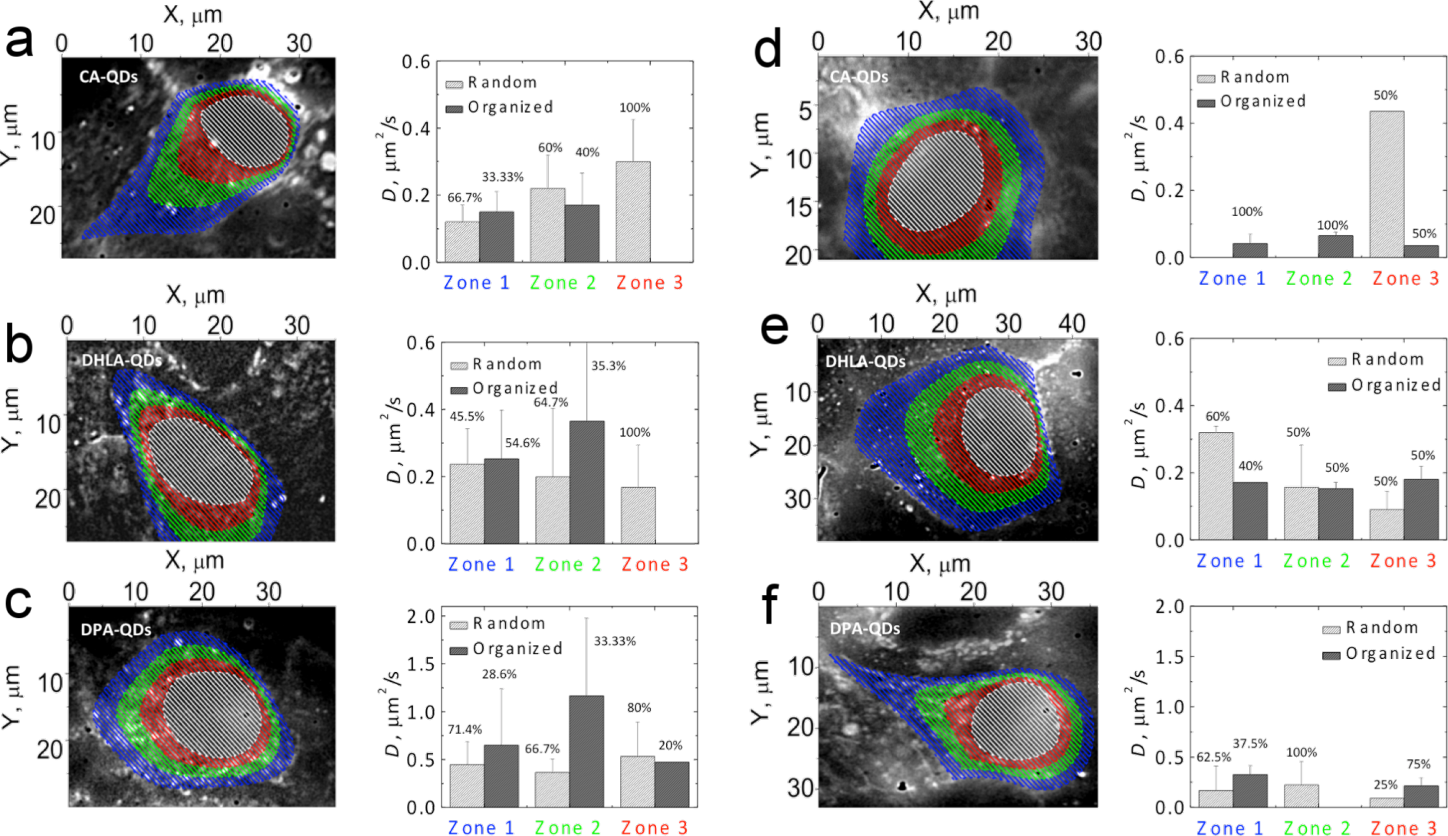
Wide-field microscopy images and corresponding diffusion constants of 10 nM solutions of CA–QDs (a,d); DHLA–QDs (b,e); and DPA–QDs (c,f) added to MDCKII cells for 3.5–4 h (a–c) and 22–23 h (d–f). Each image is an overlay of 200 consecutive frames taken with a EM-CCD camera with 0.2 s acquisition times. The highlighted areas divide the shortest distance between the nucleus and plasma membrane in three equal parts.

The behavior of QDs in different regions of MDCKII cells after 4 and 22 hours of spontaneous interaction was further investigated by tracking the movement of the nanoparticles within the cell in different areas as explained below. A series of image sequences of cells exposed to QDs with different types of surface coatings was acquired by an EM-CCD camera with 0.2 s exposure time. Then, the trajectories of fluorescent spots corresponding to moving QDs were extracted using the ImageJ plugin SpotTracker developed by Sage et al. [36] and the diffusion coefficients, *D*, were calculated from the slope of the mean square displacement (MSD)–time lag plots [37]. Here, we employed a simplified approach to roughly assign the position of tracked QDs without labeling of the cells. In order to estimate the spatial location of the QDs within the cells, we divided the cellular interior between the plasma membrane at the cell–cell contact site and the nuclear envelope, which could be identified through the corresponding transmission images as three washer-shaped circular sections as shown in Figure 5. Each section has an extension of 1/3 of the shortest distance between the plasma- and nuclear-membrane for a given point of the cellular interior. Such a classification allowed for localizing at least some typical cellular compartments. The first section or zone corresponds to the membrane and membrane-enclosed organelles; the middle zone stretches deeper within the interior of the cell, encompassing organelles such as centrioles and the Golgi apparatus; the third zone includes the nucleus-proximate region with the nuclear membrane, ribosomes and endoplasmatic reticulum.

In Figure 5, typical frame overlays for the CA-, DHLA- and DPA-coated QDs internalized into different zones of MDCKII cells after 4 and 22 h of interaction are presented along with the diffusion coefficients derived from the MSD–time lag plots. According to the character of the motion, all trajectories were divided into organized or random categories, and averaged for each zone (Figure 5). Organized zones comprise trajectories of particles that had travelled a significant distance: short (with back-and-forth contour-like movement) and long (directed motion of QDs, supposedly being dragged by motor proteins); random trajectories were assigned to disordered motion. For both trajectory types, we observed two modes of confined motion in the MSD plots of QDs inside the cellular interior. We use the fast component (typically the first 5–6 data points) to calculate the diffusion coefficient.

In the early stages of interaction (4 h after addition), the mobility of particles taken up by the cells was lowest in case of positively-charged QDs (CA–QDs) with *D* values of 0.1–0.4 μm^2^/s. More active movement was found deeper in the cellular interior, in zones 2 and 3, as compared to the membrane-enclosed zone 1 (Figure 5a). Notably, only 30–40% of QDs in zones 1 and 2 displayed organized movement, while the others diffused randomly, which was entirely true for the particle behavior in zone 3 (Figure 5a). Compared to amine-functionalized CA–QDs, carboxylated DHLA–QDs showed similar behavior in the nucleus-proximate area and slightly more mobility (*D* = 0.16–0. 52 μm^2^/s) and a more organized motion in zones 1 and 2 (Figure 5b). Finally, internalized, zwitterionic, DPA-coated QDs showed the fastest motion in all cellular compartments with *D* values ranging from 0.4 to 1.7 μm^2^/s (Figure 5c). DPA–QDs that exhibited organized motion (≈30% of the overall amount) demonstrated diffusion constants considerably larger than those randomly diffusing (Figure 5c).

After 22 h of exposure, the increased fraction of internalized particles that showed organized motion exhibited reduced mobility compared to the early stage (Figure 5d–f). This might be explained by binding of QDs to the inside or the outside of cellular compartments, which reduces the number of freely-moving QDs, and more intensively confines their movement. The random movement of the CA–QDs was observed only for very large spots, which were thus discarded. For DHLA- and DPA-coated QDs, many more QDs were found that were moving in close proximity to the nuclear envelope. Similar to earlier findings on the interaction kinetics (as shown in Supporting Information File 1, Figure S2) for DHLA–QDs, we also observed some particles in the nuclei. In the overlay presented in Figure 5e, fluorescent signals from immobile QDs were detected in nucleoli, suggesting that some small fraction of carboxylated DHLA–QDs also enter the nucleus.

**Figure 6:**
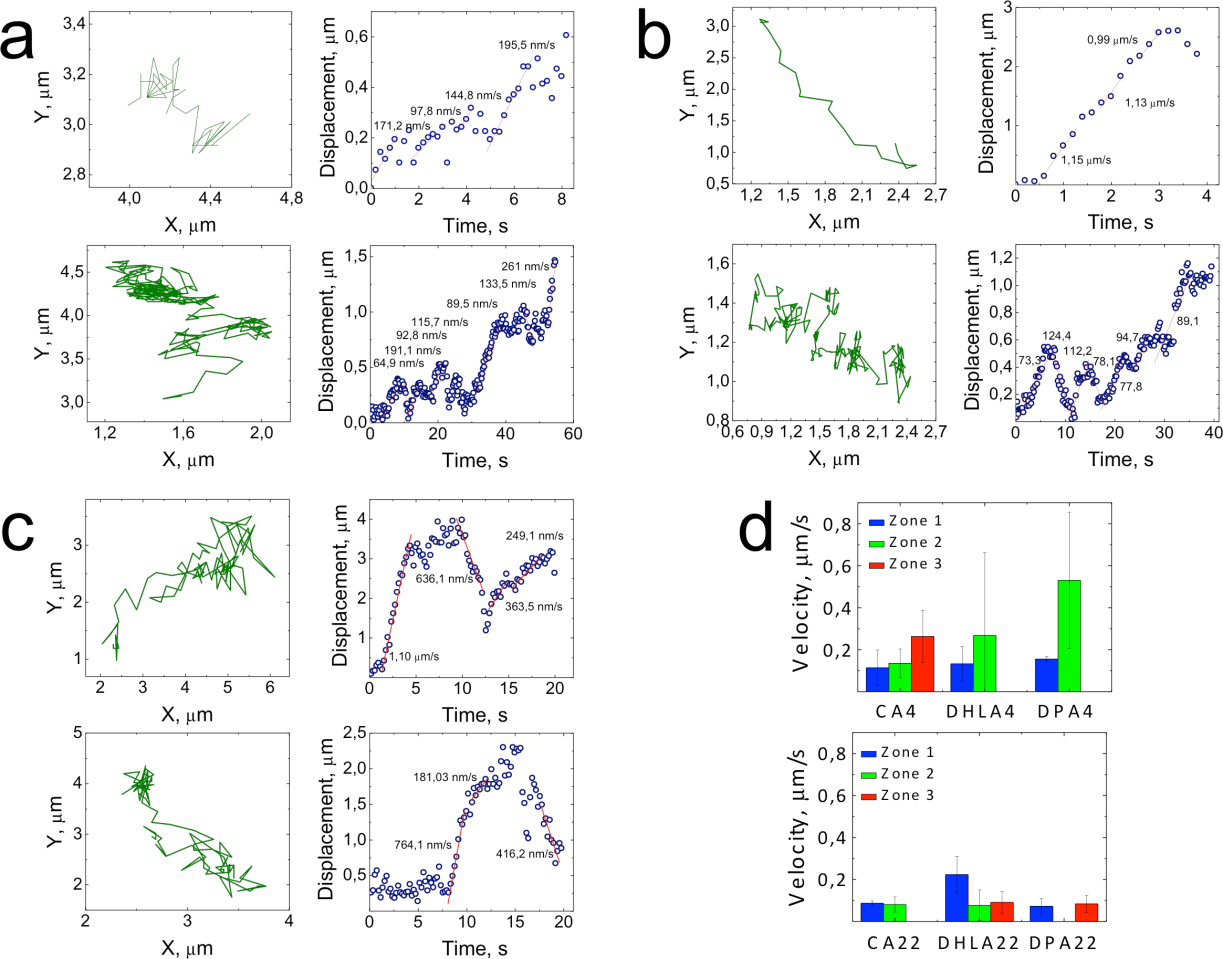
Types of organized movement observed in MDCKII cells after 4 h of exposure to CA–QDs (a), DHLA–QDs (b) and DPA–QDs (c). Each row shows the trajectory and the corresponding displacement vs time representation. (d) Average velocities of CA–QDs, DHLA–QDs and DPA–QDs exhibiting organized movement in different zones of cellular interior after 4 h (top) and 22 h (bottom) of QD incubation.

For further investigation of QDs demonstrating organized motion, we calculated the velocities of the directed phases of motion. Figure 6a–c shows various types of organized motion observed for different QD samples in zones 2 and 3 of the cellular interior after 4 h of exposure. Displacements calculated from the trajectories (green lines) were plotted as a function of time (blue circles), and the velocities for the directed modes of motion were obtained from the linear fits (red lines) (Figure 6a–c). Most of the tracked particles moved inhomogeneously, with alternating directed phases, most likely corresponding to QD or QD-contained vesicles being transported by a motor protein along cytoskeletal filaments, and nondirected phases, during which the connection between QDs and filaments was lost. The presence of such trajectories for QD–kinesin constructs in HeLa cells was previously attributed to the detaching and reattaching of kinesin molecules to microtubules [38]. We also observe back-and-forth motion along the same trajectories with similar velocities for both directions, implying that the QDs did not drift back during those phases, but were actively pulled (Figure 6a–c). For internalized peptide-coated QDs, an involvement of more than one motor protein such as kinesin or dynein was reported earlier. Here, some repetitive back-and-forth movements were assigned to the competition between motors with different directionality or conjunction of the cargo in the cytoplasm [31]. Due to the long exposure times used in our experiments (0.2 s), it was not possible to recognize single steps of the motor protein, which typically occur within 1–10 ms, or faster [31].

In Figure 6d, we present the velocities of CA-, DHLA- and DPA-coated QDs at both 4 and 22 h of interaction with MDCKII cells, averaged over the corresponding zones. Even at early stages of interaction, the directed motion seemed to be faster in zones 2 and 3 as compared to the membrane-enclosed areas. After 4 h of incubation with cells, DPA–QDs showed the fastest directed motion with an average velocity of υ = 530 nm/s in the middle section (zone 2) of the MDCKII interior. This correlates with the average velocity observed in recent reports for the movement of QD–kinesin conjugates along microtubules (in vivo in HeLa cells, υ = 500 nm/s; in vitro on crowded microtubules, υ = 560 nm/s) [38–39], and myosin V–QD constructs along actin filaments (υ = 500–600 nm/s) in living HeLa [40] and COS7 [41] cells, which were faster than the in vitro characteristics of myosin V (υ = 200–450 nm/s) [42–43]. The difference in velocities of the observed directed motion for various QD samples could be caused by the difference in size of the QD-containing vesicles (Figure 6b). Thus, internalized amine-functionalized CA–QDs were seemingly the largest cargos transported in the cellular interior, while carboxylated DHLA-coated and zwitterionic DPA-coated QDs were appreciably smaller, possibly internalized as single QDs or very small aggregates. Upon 22 h of exposure of MDCKII cells to QDs, the velocities of particles exhibiting organized motion in the cellular interior were considerably reduced to υ = 70–90 nm/s (Figure 6d), suggesting a size increase of the QD-containing vesicles.

**Figure 7:**
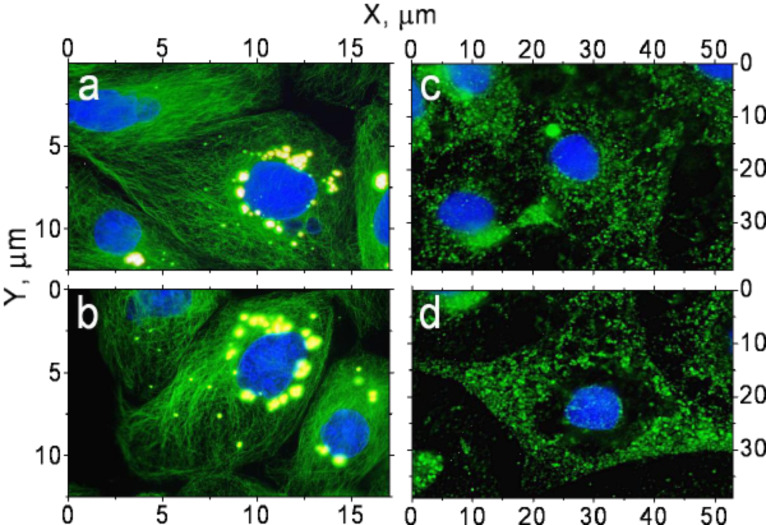
Fluorescent micrographs of untreated (a,b) and pre-incubated with 100 µM nocodazole (c,d) MDCKII cells exposed to 50 nM solution of DPA–QDs for 24 h (blue channel: DAPI-stained nuclei; green channel: Alexa Fluor 488-stained microtubules, QDs).

Figure 7 and Figure S1 of Supporting Information File 1 compare the distribution of DPA–QDs taken up after 24 h of exposure by untreated MDCKII cells and cells pre-incubated with 100 μM nocodazole in order to disrupt the microtubule network. For the untreated cells, the major fraction of QD uptake is accumulated in the perinuclear region, appearing as large bright spots in the fluorescent micrographs (Figure 7a,b). Upon nocodazole treatment, the QDs were distributed rather randomly over the cellular interior, with almost no uptake in the area close to the nucleus (Figure 7c,d). This confirms the assumption that a large amount of QDs taken up by MDCKII cells is transported along microtubules.

## Conclusion

Surface functionalization of QDs plays an important role in their nonspecific interaction with cells, determining how and in which form they enter the cellular interior. Positively-charged CdSe/ZnS QDs were found to be least cytotoxic, while negatively-charged zwitterionic QDs reduced the mitochondrial activity of MDCKII cells by up to 5 and 25%, respectively, where the cellular impedance was assessed by ECIS. The mechanism of QD-induced damage was ascertained to be more complex, rather than strictly the release of cadmium ions from CdSe cores. Pretreatment of cells with pharmacological inhibitors suggested that the uptake of nanoparticles was largely due to receptor-independent endocytosis or spontaneous entry for carboxylated and zwitterionic QDs, while for amine-functionalized particles, formation of vesicles was observed. The effect of surface charge was also observed for random and organized motions of internalized particles in the cellular interior, with both the diffusion coefficient and the velocity increasing in the following order: CA–QDs, DHLA–QDs, DPA–QDs. This result could be attributed to a decrease of the vesicle size in the same sequence. Thus, combining these observations with cytotoxicity characteristics (which increase in the same order), we can conclude that surface functionalization stipulates the conditions under which QDs are internalized into cells. It appears that the smallest vesicles/aggregates or even single particles tend to induce the most damage.

In addition, we demonstrate that unlike the conventional MTS assay used for the assessment of vitality, monitoring the impedance response by the ECIS technique allows detection of the presence of QDs in cells and evaluation of their impact, even at early stages. This work highlights the importance of the combined use of ECIS and MTS (or other biochemical) assays for the characterization of nanoparticle cytotoxicity.

## Experimental

### Cell culture

MDCKII cells were maintained in Earle’s minimum essential medium supplemented with 4 mM glutamine, 100 g/mL of both penicillin and streptomycin (Biochrom, Berlin, Germany), 10% (v/v) fetal calf serum (PAA Laboratories GmbH, Cölbe, Germany) and stored in incubators (HERA cell 150, Heraeus, Germany) with a 5% CO_2_ atmosphere. Cells were subcultured weekly after reaching confluence by washing with PBS, followed by trypsinization and centrifugation at 110*g*. Counting was carried out using a Neubauer chamber, and viability was determined using trypan blue exclusion. For fluorescence microscopy measurements, the cells were grown in 2 mL of cell culture medium in a ibiTreat µ-Dish (Ibidi, Martinsried, Germany) for 48 h.

### Immunostaining

After washing with PBS, fixation was carried out by immersing the cells into a 20 °C cold acetone/methanol mixture (1:1 v/v) for 10 min. Afterwards, the cells were washed three times with PBS, the unspecific binding sites were blocked with FCS, and incubation in staining solutions was carried out according to the manufacturer’s recommendation: Alexa Fluor-conjugated IgG1 anti-tubulin (BD Bioscience, Heidelberg, Germany) from mouse was used for labeling microtubules, and 4’,6-diamidino-2-phenylindole (DAPI, Sigma-Aldrich, Seelze) for nucleus and DNA labeling. Staining was carried out for 30 min at room temperature, cells were then washed and taken to a microscope.

### Inhibition of endocytosis

MDCKII cells were pretreated with 25 μg/mL chlorpromazine [44], 30 μg/mL 5-(*N*,*N*-dimethyl)amiloride [45] and 1 μg/mL filipin III [46] (predissolved in dimethyl sulfoxide 0.5 μg/μL) upon incubation at 36.6 °C in culture medium (prepared as described above) for 30 min, 15 min and 1 h, respectively. The cells were subsequently washed with cell culture medium and taken to the microscope for experiments. All inhibitors were purchased from Sigma-Aldrich at highest purity grades available.

### CdSe core synthesis

The synthesis of the CdSe core of the quantum dots and the ZnS shell followed a modified prescription from Mahler et al. [47] The Se injection solution was prepared by heating 64 mg of Se, 4 mL of trioctylphosphine, 1.5 mL of oleylamine and 1 g of tetradecylphosphonic acid under argon flow until the solution became clear. In a separate three-neck flask, 102.8 mg of CdO, 2 mL of oleic acid and 3 mL of 1-octadecene were mixed and degassed under vacuum at 70 °C for 1 hour. Then the system was switched to an argon atmosphere and the temperature was increased to 240 °C for the injection of Se solution. The nanocrystals were left to grow for 6 minutes at 200 °C, then the heating was removed and the flask was cooled down to ≈80 °C by compressed air flow before the addition of 40 mL of ethanol. The sample was centrifuged at 4000 rpm for 5 minutes; the resulting pellet containing CdSe QDs was redissolved in 10 mL of hexane and sonicated for 5 minutes in order to purify the sample from the excess of surfactants. The clear supernatant was precipitated again with ethanol and centrifuged for another 5 minutes. Finally, the purified nanoparticles were redissolved in chloroform.

### Coating with ZnS

A 0.1 M Zn stock solution was prepared by dissolving 632.3 mg of zinc stearate in 2 mL of oleic acid and 7.5 mL of oleylamine, while a 0.1 M S stock solution was obtained by mixing 32 mg of elemental sulfur in 10 mL of 1-octadecene. Both solutions were prepared at 200 °C under argon flow.

In a three-neck flask, the CdSe cores (typically 2 × 10^17^ nanoparticles), 2 mL of oleylamine and 10 mL of 1-octadecene were degassed under vacuum at 70 °C for 30 minutes. Then the flask was filled with argon, and the amount of S stock solution corresponding to a single monolayer was added to the reaction mixture under vigorous stirring. After 15 minutes at 175 °C, the Zn stock solution was added to the completed first monolayer of ZnS. The temperature was further increased to 220 °C and 0.1 M stock solutions of Zn and S were injected dropwise in an alternating manner in 15 min intervals. The amount of Zn and S injection solutions necessary for the growth of each monolayer of ZnS were calculated from the bulk densities of CdSe and ZnS and the CdSe core diameter (determined by TEM). After the last injection, the system was left to react for 25 minutes, then the heat was removed and the mixture was cooled down to room temperature with compressed air flow. The resulting core/shell QDs were precipitated with ethanol, centrifuged and redissolved in chloroform.

### QD surface functionalization

Table 1 summarizes the amounts of ligands and solvents used for QD surface functionalization.

**Table 1:** Ligands and solvents used for QD functionalization.

	CA	DHLA	MPA	DPA

Ligand	57 mg	92 μL	44 μL	75 mg
Solvent	2-propanol, 20 mL	1:1 methanol/dioxane, 20 mL	1:1 methanol/dioxane, 20 mL	2-propanol, 20 mL
TMAHP	-	pH ≈12	pH ≈12	pH ≈11

The amount of desired ligand was dissolved in 20 mL of solvent, the pH was adjusted upon the addition of tetramethyl ammoniumhydroxide pentahydrate (TMAHP) (if required), and the mixture was heated to 70 °C upon vigorous stirring under argon flow. After the temperature was reached, 0.2 mL of 100 μM CdSe/ZnS QD stock solution in chloroform was added to the refluxing solution and stirred for 10–15 minutes. Subsequently, the mixture was cooled down by compressed air flow to RT and ethyl acetate was added to precipitate the ligand-exchanged QDs. The sample was then centrifuged for 40 min at 4000 rpm and the resulting pellet was redissolved in 1 mL of distilled water.

### Fluorescence microscopy measurements

For the fluorescent staining experiments, an upright Olympus fluorescence microscope Olympus BX51, with a 40× water-immersion objective (Olympus, Tokyo, Japan), equipped with a color camera (3 MP) was used. The kinetics of the nonspecific interaction between MDCKII cells and QDs was measured using a Zeiss Axiovert 135 TV epifluorescence microscope (Carl Zeiss GmbH, Oberkochen, Germany) with a 100×, oil-immersion, plan, apochromat objective (Carl Zeiss GmbH, Oberkochen, Germany). Excitation light (458–490 nm) was delivered from a mercury lamp and the emission light (497–567 nm) was detected by a LaVision CCD camera (LaVision GmbH, Goettingen, Germany). For the single-particle tracking experiments, a homebuilt, wide-field microscope with a 100×, oil-immersion, plan, apochromat objective (Carl Zeiss GmbH, Oberkochen, Germany) was used. In this case, the excitation light was delivered from an Ar^+^ gas laser operating at 488 nm and the emission light detected by a sensitive Andor iXon EM-CCD camera (Andor Technology, Belfast, Northern Ireland).

MDCKII cells were maintained at physiological conditions throughout the experiments in a miniature incubator (BioScienceTools, San Diego, USA), at 36.6 °C with a supply of humidified, 5% CO_2_ air–gas mixture. We used the same initial CdSe/ZnS core/shell QD sample (4 monolayers) to prepare different surface functionalization for fluorescence microscopy measurements and single-particle tracking (emission maximum 529 nm, FWHM = 36 nm).

### ECIS measurements

For the measurements, approximately 150,000 MDCKII cells were seeded onto gold electrodes of 96W1E arrays (Ibidi, Martinsried, Germany) suspended in 200 µL of culture medium and stored in an incubator set to 37 °C and 5% CO_2_. After 24 h of adhesion and spreading of the cells, the cell layer reached confluency and was treated with QDs carrying different surface modifications. Time-resolved impedance data were acquired with the ECIS setup ECIS ZΩ (Applied Biophysics, Troy, NY). Impedance background data were recorded at a frequency of 4 kHz [25–27,48–50].

## Supporting Information

File 1 Additional figures comprising fluorescence micrographs and tracks.
