# Supplementary material for: The effect of surface charge on nonspecific uptake and cytotoxicity of CdSe/ZnS core/shell quantum dots
Source: Beilstein J Nanotechnol. 2015 Jan 26;6:281–92. doi: 10.3762/bjnano.6.26 (PMC4362492; doi:10.3762/bjnano.6.26)
Supplement: File 1 — Additional figures comprising fluorescence micrographs and tracks. [file Beilstein_J_Nanotechnol-06-281-s001.pdf]

Supporting information

for

The effect of surface charge on nonspecific uptake and cytotoxicity of CdSe/ZnS core/shell quantum dots

Vladimir V. Breus[1], Anna Pietuch[2], Marco Tarantola[3], Thomas Basché*[1] and Andreas Janshoff*[2]

Address: [1]Institute of Physical Chemistry, Johannes Gutenberg University Mainz, Jakob-Welder-Weg 11, 55128 Mainz, Germany, [2]Institute of Physical Chemistry, University of Goettingen, Tammannstr. 6, 37077 Goettingen, Germany and [3]Max-Planck-Institute for Dynamics and Self-Organization (MPIDS), Laboratory for Fluid Dynamics, Pattern Formation and Biocomplexity, Am Fassberg 17, 37077 Goettingen, Germany

Email: Andreas Janshoff - ajansho@gwdg.de; Thomas Basché - basche@uni-mainz.de

* Corresponding author

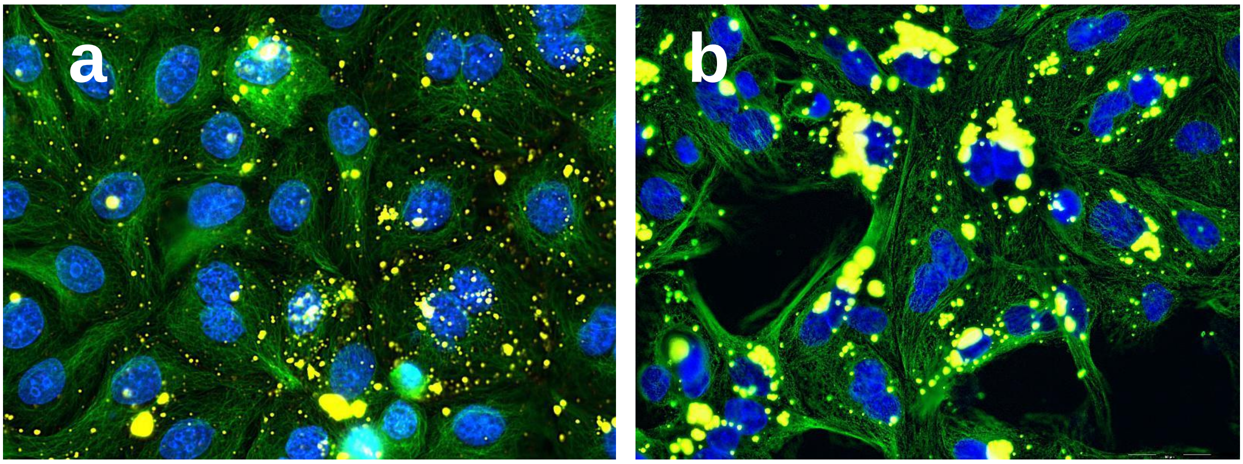

Figure S1: Fluorescence micrographs of MDCKII cell layer regions containing overgrown 2-nuclei cells as a result of 24 h exposure to 50 nM solutions of (a) MPA-coated and (b) DPA-coated CdSe/ZnS QDs (blue channel: DAPI-stained nuclei; green channel: Alexa Fluor 488-stained microtubules; yellow channel: QDs).

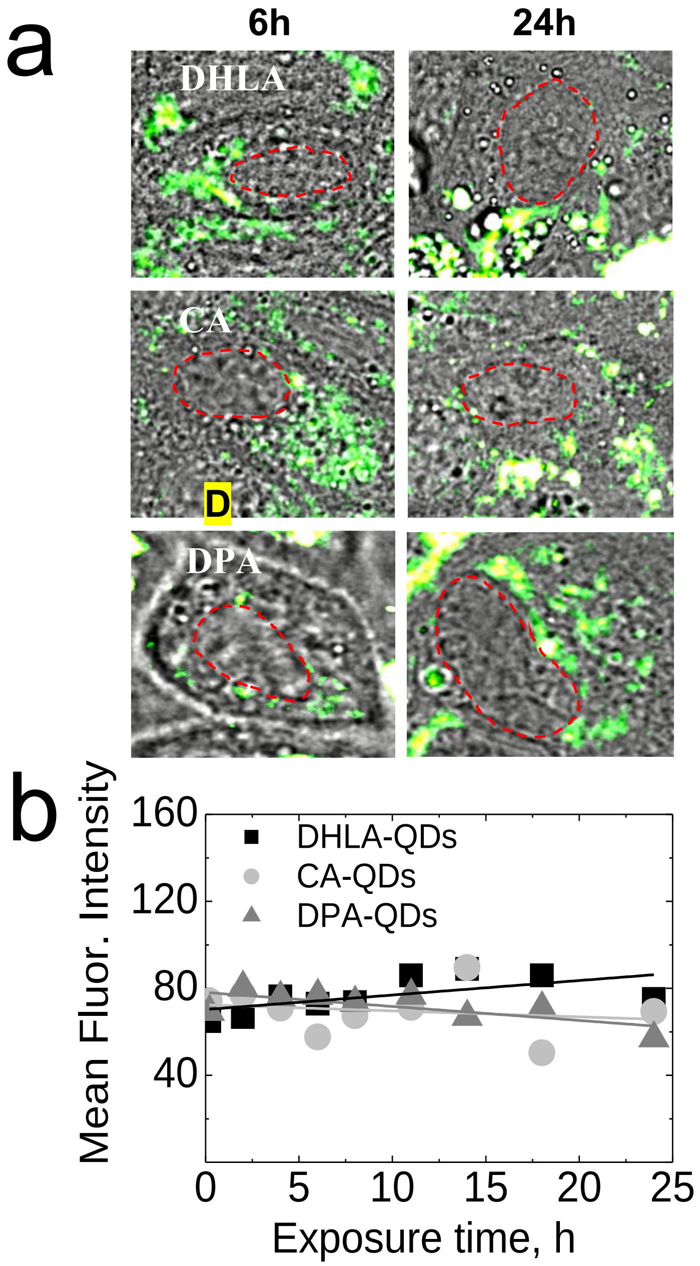

Figure S2: (a) Overlay of fluorescence with transmission images of CA-, DHLA-, CA- and DPA-functionalized CdSe/ZnS quantum dots taken at 6 and 24 h after exposure to MDCKII cells. (b) Mean fluorescence intensity in the nuclei of MDCKII cells upon 24 hours of interaction with CA-, DHLA-, and DPA-coated QDs. Straight lines are linear fits.

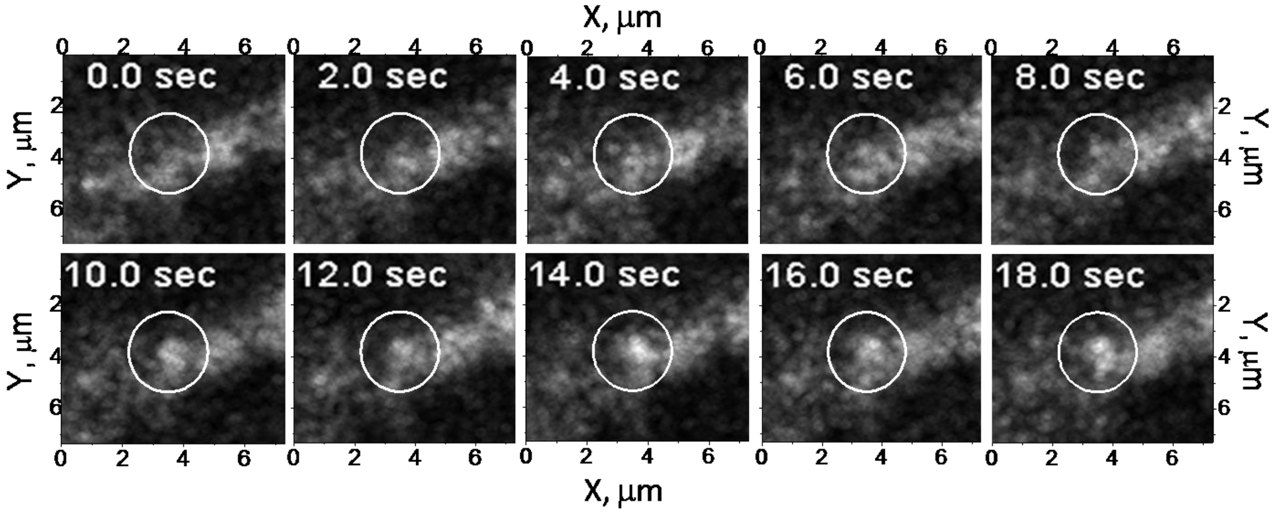

Figure S3: Formation of QD-containing vesicles from the MDCKII plasma membrane after 2 h of exposure to CA–QDs.

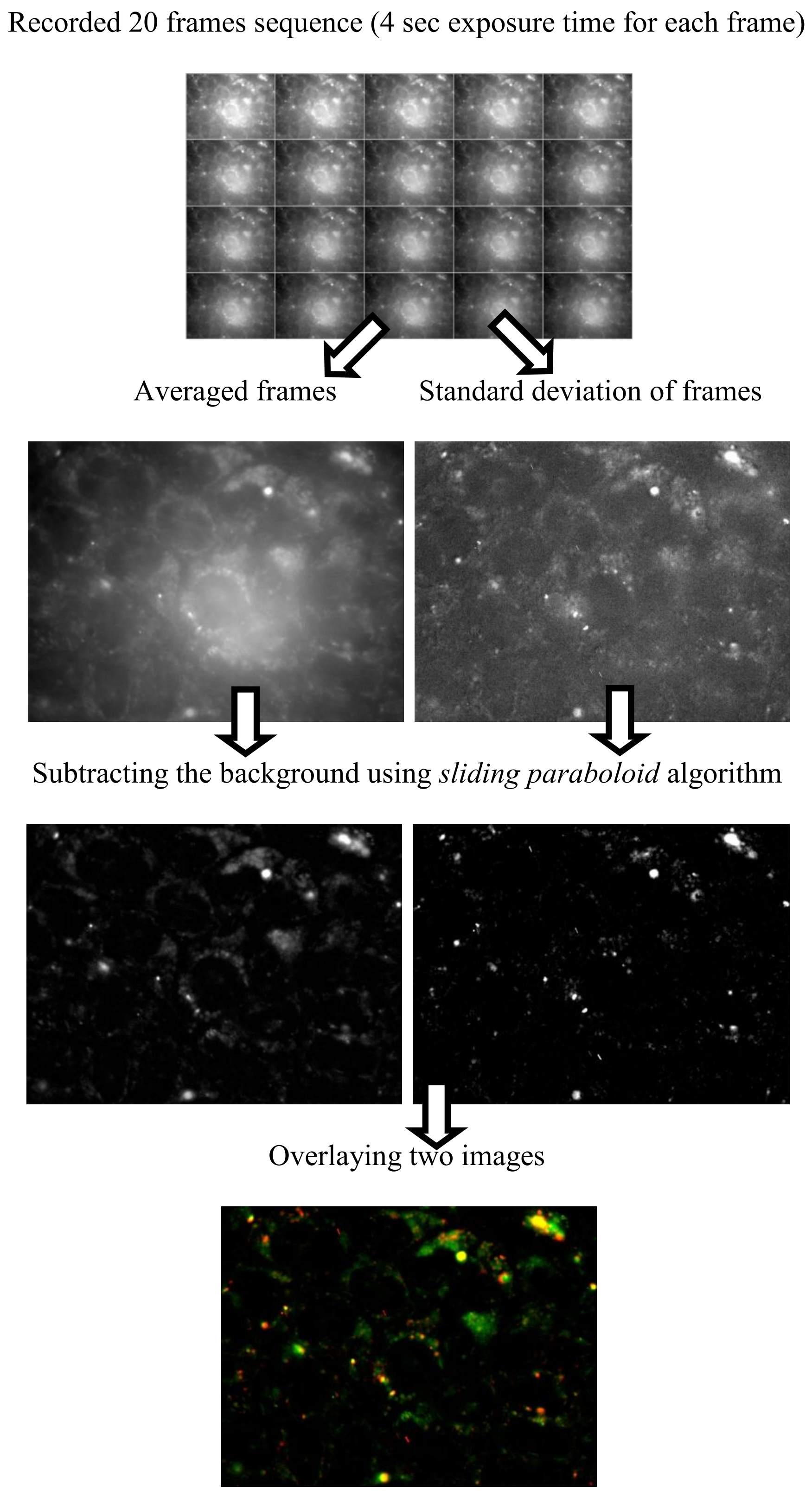

Figure S4: Image post-processing. Standard deviation of a frame sequence contain only fluorescent spots which change their position during 80 s (20 × 4 s) scan, while the averaged signal contains both QD and amplified cell fluorescence. All image processing was performed with ImageJ 1.40g open source software (Wayne Rasband, National Institutes of Health, USA).
